# In Vivo Characterization of a Bank Vole-Derived Cowpox Virus Isolate in Natural Hosts and the Rat Model

**DOI:** 10.3390/v12020237

**Published:** 2020-02-20

**Authors:** Saskia Weber, Kathrin Jeske, Rainer G. Ulrich, Christian Imholt, Jens Jacob, Martin Beer, Donata Hoffmann

**Affiliations:** 1 Institute of Diagnostic Virology, Friedrich-Loeffler-Institut, Federal Research Institute for Animal Health, Südufer 10, 17493 Greifswald-Insel Riems, Germany; saskia.weber@fli.de (S.W.); kathrin.jeske@gmx.de (K.J.); 2 Institute of Novel and Emerging Infectious Diseases, Friedrich-Loeffler-Institut, Federal Research Institute for Animal Health, Südufer 10, 17493 Greifswald-Insel Riems, Germany; rainer.ulrich@fli.de; 3 Vertebrate Research, Institute for Plant Protection in Horticulture and Forests, Julius Kühn-Institute, Toppheideweg 88, 48161 Münster, Germany; christian.imholt@julius-kuehn.de (C.I.); jens.jacob@julius-kuehn.de (J.J.)

**Keywords:** cowpox virus (CPXV) *Orthopoxvirus*, bank vole (*Myodes glareolus*), common vole (*Microtus arvalis*), reservoir host, animal model

## Abstract

Cowpox virus (CPXV) belongs to the genus *Orthopoxvirus* in the *Poxviridae* family and is endemic in western Eurasia. Based on seroprevalence studies in different voles from continental Europe and UK, voles are suspected to be the major reservoir host. Recently, a CPXV was isolated from a bank vole (*Myodes glareolus*) in Germany that showed a high genetic similarity to another isolate originating from a Cotton-top tamarin (*Saguinus oedipus*). Here we characterize this first bank vole-derived CPXV isolate in comparison to the related tamarin-derived isolate. Both isolates grouped genetically within the provisionally called CPXV-like 3 clade. Previous phylogenetic analysis indicated that CPXV is polyphyletic and CPXV-like 3 clade represents probably a different species if categorized by the rules used for other orthopoxviruses. Experimental infection studies with bank voles, common voles (*Microtus* *arvalis*) and Wistar rats showed very clear differences. The bank vole isolate was avirulent in both common voles and Wistar rats with seroconversion seen only in the rats. In contrast, inoculated bank voles exhibited viral shedding and seroconversion for both tested CPXV isolates. In addition, bank voles infected with the tamarin-derived isolate experienced a marked weight loss. Our findings allow for the conclusion that CPXV isolates might differ in their replication capacity in different vole species and rats depending on their original host. Moreover, the results indicate host-specific differences concerning CPXV-specific virulence. Further experiments are needed to identify individual virulence and host factors involved in the susceptibility and outcome of CPXV-infections in the different reservoir hosts.

## 1. Introduction

The zoonotic Cowpox virus (CPXV), currently a single species within the genus *Orthopoxvirus*, is endemic in Europe and northern and central Asia [1,2]. Many mammals are known to be susceptible to CPXV infection and disease, like cats (*Felis catus*) [3,4], pet rats (*Rattus norvegicus forma domestica*) [5,6,7], alpacas (*Vicugna pacos*) [8], Asian elephants (*Elephas maximus*) [9], and primates [10,11]. Maintenance of viral infections within these species is not achieved and therefore these species represent accidental hosts for the virus [12]. Spill-over infections from these accidental hosts to humans happen frequently, mostly through micro skin lesions like scratches or abrasions. The resulting human infections are generally characterized by a mild and self-limiting illness with typical pox lesions at the site of virus entry [3,6,7,13]. However, the virus can also cause systemic and fatal disease in immuno-compromised patients [14,15]. Routine vaccination against smallpox—which supposedly provides protection against CPXV-induced disease—ended in the 1970s. Today, more than half of the world’s human population is unvaccinated [16,17], due to the cessation of smallpox vaccination, and the number of human CPXV infections have increased [18].

In contrast to an accidental host, a reservoir could be defined as “a population which is chronically infested with the causative agent of a disease and can infect other populations” [19]. A reservoir host in particular could be “one or more epidemiologically connected populations or environments in which the pathogen can be permanently maintained and from which infection is transmitted to the defined target population” [20].

For CPXV, wild rodents are thought to be reservoir hosts in different parts of Eurasia. In Turkmenistan and Georgia, the virus was detected in a very low prevalence in wild ground squirrels (yellow suslicks; *Citellus fulvis*) and gerbils (*Rhombomys opimus*, *Meriones libycus,* and *M. meridianus*) [21,22]. In Western Europe, the highest prevalence of orthopoxvirus (OPV) reactive antibodies was detected in bank voles (*Myodes glareolus*), field voles (*Microtus agrestis*), and wood mice (*Apodemus sylvaticus*). However, no virus isolation was successful until recently [23,24], and the DNA detection rate in common voles (*Microtus arvalis*) and bank voles was very low [25,26].

In 2015, the first molecular detection and isolation of CPXV from a putative reservoir host, the common vole, was described by our group [27]. In vitro and in vivo studies with the common vole-derived isolate CPXV FM2292 (phylogenetically classified as CPXV-like 2, [28]) confirmed the reservoir competence of this vole species. Virus shedding was detectable in all inoculated animals and viral titers in swab samples reached up to 10^4^ tissue culture infectious doses 50% (TCID_50_) mL^−1^. The animals had to be euthanized because of their deteriorating health. Norway rats (*Rattus norvegicus*), strain Wistar, representing an established animal model for CPXV [29,30], were also intranasally inoculated with CPXV FM2292. They exhibited even more prominent respiratory clinical signs than common voles [27]. In addition, experimental studies with the other putative reservoir host, the bank vole, were performed [31]. However, independent of the application route or the CPXV isolate used (different phylogenetic clades and host origin) or the bank vole evolutionary lineage, viral shedding was not detectable. Consequently, the reservoir competence of bank voles for CPXV was put into question. Nevertheless, a bank vole-derived isolate for experimental proof was not available at this time.

That changed in 2017 and 2018, when bank voles were trapped in Thuringia, Germany and 509 nasal septa were screened for OPV-DNA via quantitative PCR (qPCR) [32]. Five samples tested positive for OPV-DNA, and one bank vole-derived virus isolate (CPXV GerMygEK 938/17) could be obtained. Phylogenetic analyses demonstrated a nucleotide sequence identity of 99.2% to another German CPXV isolate, Ger2010MKY (CPXV-like 3, [28]), and a clustering together with Ectromelia virus (ECTV), and a separation from other CPXV isolates derived from either common voles or accidental host species. Viral replication on the chorioallantoic membrane (CAM) resulted in hemorrhagic pocks typical for CPXV [33], whereas ECTV develops non-hemorrhagic pocks on the CAM [34]. The taxonomical species definition for other orthopox viruses conceded the presumption that the different clades “CPXV-like 1”, “CPXV-like 2”, and “CPXV-like 3” would be better classified as different species within the genus *Orthopoxvirus*.

The closest relative CPXV strain Ger2010MKY was isolated during a fatal outbreak among cotton-top tamarins (*Saguinus oedipus*) in a Thuringian animal park in 2010 [10]. All infected cotton-top tamarins died peracutely, while common marmosets (*Callithrix jacchus*) in the same husbandry remained healthy, but scored qPCR positive in swab samples [10]. Whether bank voles or other rodents were involved in the history of the diseased cotton-top tamarins in 2010 remained unclear. The isolate CPXV Ger2010MKY was tested in Wistar rats for assessment of viral pathogenicity according to established protocols [29,30]. Rats were inoculated intranasally using 10^4^ or 10^5^ TCID_50_ mL^−1^. All collected buccal swabs were OPV DNA negative and only one animal out of four per dosage group seroconverted [10]. 

Here we describe the first in vivo characterization of the bank vole-derived CPXV strain “GerMygEK 938/17”. Comparative infection experiments were performed using (i) bank voles, as the putative original reservoir host, (ii) common voles as an additional potential reservoir host, and (iii) Wistar rats as a surrogate animal model. The closely related Ger2010MKY isolate was also included to prove if strains of the same Ectromelia-like clade—provisionally named “CPXV-like 3”—behave similarly [28]. 

These experiments revealed for the first time the detection of viral shedding by CPXV-infected bank voles, while the Wistar rats only seroconverted and the common voles exhibited no indication of detectable viral replication. 

## 2. Materials and Methods 

### 2.1. Viruses and Cell Lines

CPXV strain GerMygEK 938/17 was isolated from the nasal septum of a bank vole collected during the project “Population dynamics of rodent hosts of zoonotic disease: interaction of climate, landuse and biodiversity” on a forest plot in the federal state of Thuringia, Germany, in spring 2017 [32]. CPXV Ger2010MKY has been isolated from cutaneous lesions of cotton-top tamarins housed in a animal park in Thuringia, Germany and has been already characterized by experimental inoculation of Wistar rats before [10]. As reference strains and controls for in vitro characterization, the CPXV laboratory reference strain Brighton Red and the CPXV isolate RatPox09, as representative of a highly virulent strain in Wistar rats [27] were used.

African green monkey (*Chlorocebus* spec.) cells (Vero76, Collection of Cell Lines in Veterinary Medicine CCLV, Friedrich-Loeffler-Institut, Greifswald-Insel Riems, Germany), were grown and maintained in Eagle’s minimal essential medium (MEM; Biochrom GmbH, Berlin, Germany) supplemented with 10% fetal calf serum (FCS, Biochrom GmbH, Berlin, Germany) and kept under a 5% CO_2_ atmosphere at 37 °C. 

All CPXV strains were cultivated and titrated on Vero76 cells, and stock titers of approximately 10^7^ TCID_50_ mL^−1^ were achieved.

### 2.2. In Vitro Characterization: Virus Growth Kinetics

Vero76 cells from overnight cultures were inoculated with CPXV GerMygEK 938/17 or CPXV Ger2010MKY at a multiplicity of infection (MOI) of 0.01 or 3. For reference, we used a virus strain characterized as highly virulent in Wistar rats, namely CPXV RatPox09, and laboratory strain CPXV Brighton Red. After inoculation, cells were incubated at 37°C for 60 min under a 5% CO_2_ atmosphere and were washed afterwards three times with phosphate-buffered saline (PBS). Subsequently, fresh cell culture medium (MEM supplemented with 10% FCS) was added. The first samples were collected immediately afterwards (time point 0h), and further samples from five different time points (6h, 12h, 24, 48, and 72h) were obtained (four biological replicates per sample). Virus titers were determined as technical duplicates by endpoint dilution assay on Vero76 and calculated as TCID_50_ mL^−1^ using the Spearman-Kärber algorithm [35,36]. The detection limit of our test is 10^1.625^ TCID_50_ mL^−1^. Altogether, for each biological replicate we got two technical replicates and therefore overall eight values for each virus. 

### 2.3. In Vivo Characterization: Animal Experiments and Analysis of Samples

#### 2.3.1. Animals

The animal experiments were approved by the Ministry of Agriculture of Mecklenburg-Western Pomerania, Germany, under reference number LALLF M-V 7221.3-2-004/18, 15/03/2018.

Bank voles originated from our in-house breeding. Wistar rats were purchased from Charles River Laboratories (Sulzfeld, Germany) and common voles originated from the Julius Kühn-Institute. They were housed in standard laboratory rodent cages in groups of 2 to 4 animals (rats and bank voles) or individually (common vole) under standardized conditions (22°C; 12/12h light cycle). As diet, rodent pellets (SSniff Spezialdiäten GmbH, Soest, Germany) and water ad libitum were fed. For acclimatization, rodents were housed under these conditions for one week prior to inoculation. 

All animals were outbred animals, of mixed sex and mixed age except the Wistar rats, which were all six weeks of age.

#### 2.3.2. Infection Experiments and Sampling 

The design of the animal experiments is shown in Table 1. Ten rats, six bank voles, and six common voles were inoculated with the new isolate CPXV GerMygEK 938/17. Because CPXV Ger2010MKY had been already tested in Wistar rats [10], only six bank voles and six common voles were inoculated with this isolate. All CPXV strains were applied oronasally with a dosage of 10^5.5^ TCID_50_/animal after a short isoflurane induced anesthesia. The general health status was checked daily over a period of 28 days. In addition, body weight measurement and buccal swab (Copan, Brescia, Italy) sampling (under isoflurane anesthesia) was performed every other day until day 21 post inoculation. 

The animals were euthanized 28 days post inoculation (dpi) and serum, peritoneal lavage and an organ panel (turbinate, skin, liver, lung, spleen, and trachea) were collected.

#### 2.3.3. Determination of Viral Genome Loads and Infectious Virus Titers from Buccal Swabs and Organ Samples

Buccal swab samples were resuspended in 2 mL cell culture medium (MEM) and antibiotics were added (enrofloxacin, 1%; Bayer, Leverkusen, Germany; amphotericin/gentamicin, 0.2%; Thermo Fisher Scientific Inc, Schwerte, Germany; lincomycin, 0.5%; WDT, Garbsen, Germany). The organ samples were transferred into 1 mL cell culture medium supplemented with 10% FCS and antibiotics (1% penicillin-streptomycin, Biochrom GmbH, Berlin, Germany). For mechanic homogenization (TissueLyser II; Qiagen, Hilden, Germany) all reaction tubes contained stainless steel beads (5 mm diameter; TIS Wälzkörpertechnologie GmbH, Gauting, Germany). 

Viral DNA from all buccal swabs and tissue samples was extracted by using the BioSprint 96 instrument (Qiagen, Hilden, Germany) and the NucleoMag VET kit (Macherey-Nagel, Berlin, Germany). OPV-specific DNA was detected by qPCR as described previously [37]. 

In addition, an endpoint-dilution assay was used for virus titration of the samples, and the respective data are calculated as TCID_50_ mL^−1^ using the Spearman-Kärber algorithm [35,36]. 

#### 2.3.4. Serology

Sera as well as peritoneal lavage samples were analyzed for OPV-reactive antibodies by an indirect immunofluorescence test as previously described [27]. In brief, CPXV-infected Hep2 cells (CCLV, Friedrich-Loeffler-Institut) were fixed with methanol-acetone (1:1) and incubated for 30 min at 50°C with Tris-buffered saline plus Tween (Sigma, St. Louis, MO, USA). In the following step, the cells were incubated for 1h at room temperature with a dilution series (1:20, 1:40, 1:80, 1:160, 1:320, 1:640, and 1:1280) of the inactivated (30 min at 56 °C) serum samples. After washing three times with PBS, a commercial anti-mouse or in case of the rat samples a commercial anti-rat secondary antibody conjugate (Thermo Fisher Scientific Inc, Schwerte, Germany) was applied. The cells were visualized under a fluorescence microscope (Leica DMI3000 B, Leica Wetzlar, Germany). Animals with titers of 1:40 or higher were considered positive. The titer was taken as the reciprocal of the greatest serum dilution, which showed a positive detection. Peritoneal lavage samples were handled just as the sera. 

#### 2.3.5. Figures

GraphPad PRISM (GraphPad Software, La Jolla, CA, USA) was used for generating illustrations. 

## 3. Results

### 3.1. In Vitro Characterization: Virus Growth Kinetics

**Figure 1:**
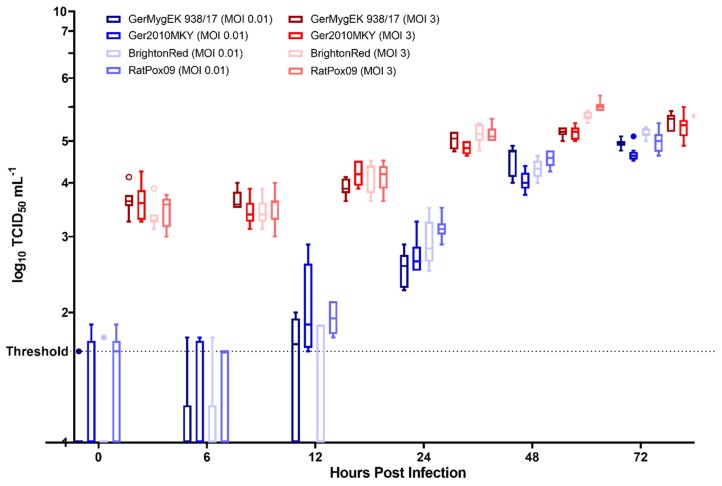
Comparison of replication characteristics of GerMygEK 938/17, Ger2010MKY, Brighton Red, and RatPox09. Vero76 cells were inoculated using multiplicities of infection (MOI)s of 0.01 and 3. Viral titers were determined by endpoint dilution assays. The mean and the standard deviations of four independent experiments, including two technical replicates per sample, are presented. The detection limit of our test was defined at 10^1.625^ TCID_50_ mL^−1^ (threshold).

CPXV GerMygEK 938/17 and CPXV Ger2010MKY were characterized on Vero76 cells in terms of their in vitro growth kinetics and compared to RatPox09, a strain highly virulent for Wistar rats, and the laboratory reference strain Brighton Red. In four independent experiments with technical duplicates as internal controls, growth kinetics of the CPXV strains were determined using different MOIs. In general, all four tested CPXV strains replicated similarly in cell culture independently from the used MOI. At 72 h post inoculation (hpi) titers of ~10^5^ TCID_50_ mL^−1^ (MOI 0.01) or ~10^6^ TCID_50_ mL^−1^ (MOI 3) were determined (Figure 1). 

### 3.2. In Vivo Characterization: Animal Experiments and Sample Analyses

For the determination of the in vivo characteristics of CPXV GerMygEK 938/17, we inoculated bank voles, common voles, and Wistar rats. In addition, bank voles and common voles were inoculated with CPXV Ger2010MKY.

#### 3.2.1. Morbidity and Mortality: Weight Loss in Bank Voles in the CPXV Ger2010MKY Group

**Figure 2:**
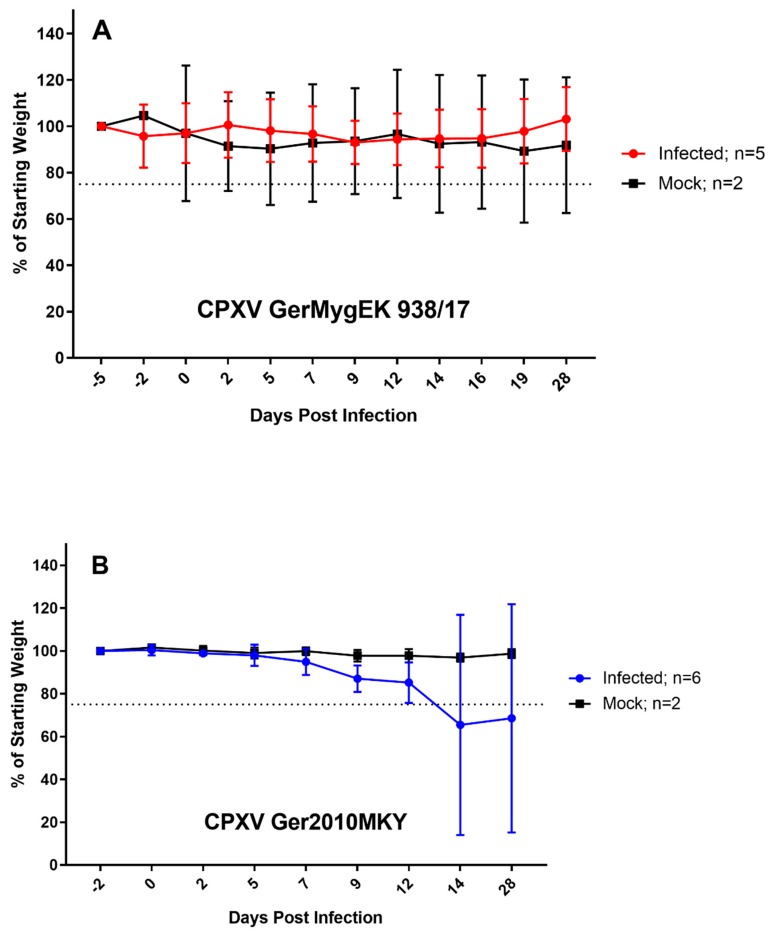
Body weight development of bank voles inoculated with (**A**) Cowpox virus (CPXV) GerMygEK 938/17 (bank vole-derived) or (**B**) CPXV Ger2010MKY (cotton-top tamarin-derived). The starting weight was set as 100%, and all values are means with standard deviations. Weight loss of more than 25% of initial weight (dotted line) was defined as termination criterion.

Oronasal inoculation with the novel bank vole-derived isolate CPXV GerMygEK 938/17 did not result in any clinical signs regardless of the inoculated species. In addition, body weights were stable for all animals for the duration of the observation period (Figure 2A). One bank vole died during inoculation (unrelated death due to anesthesia).

**Figure 3:**
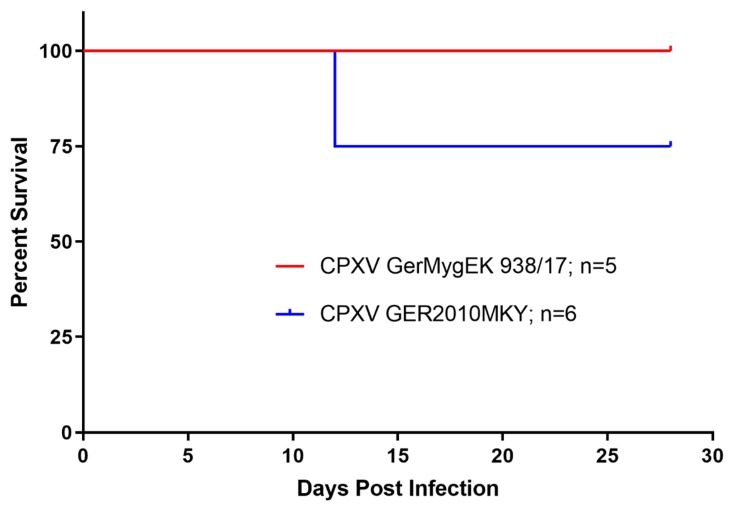
Bank vole survival rate over the time in two experiments. Bank voles were inoculated with 10^5.5^ TCID_50_ per animal of CPXV GerMygEK 938/17 or Ger2010MKY, respectively.

Following CPXV Ger2010MKY inoculation, all bank voles showed weight loss, two bank voles with up to more than 25% of their initial weight (Figure 2B), and these two animals had to be euthanized at day 12 post infection. Finally, a survival rate of 75% was recorded for the CPXV Ger2010MKY infection in bank voles (Figure 3). Obvious clinical signs were not recorded in the other bank voles.

In contrast, body weight data from common voles demonstrated weight loss in males and weight gain in females regardless of the inoculated virus (data not shown).

#### 3.2.2. Virus Shedding: Infected Bank Voles Excrete CPXV

**Figure 4:**
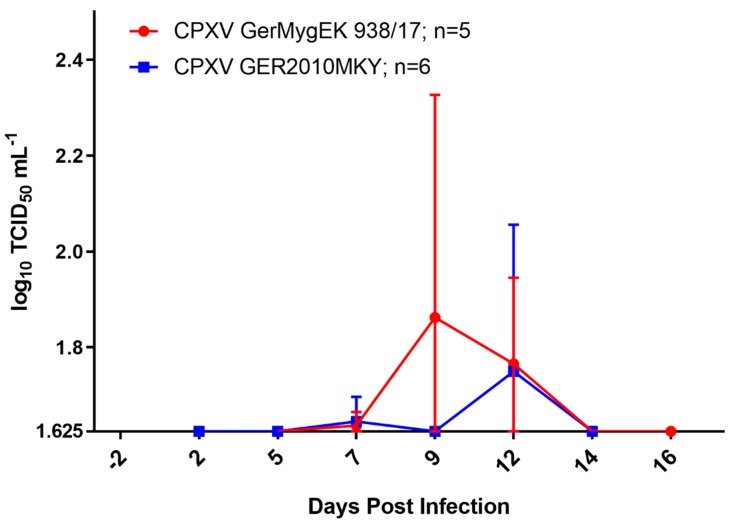
Viral shedding patterns of bank voles inoculated with CPXV GerMygEK 938/17 (red; *n* = 5) or CPXV Ger2010MKY (blue; *n* = 6) determined for buccal swabs. Values are the means with standard deviations. The detection limit of our test was defined at 10^1.625^ TCID_50_ mL^−1^.

Viral DNA could be detected in buccal swabs in each CPXV GerMygEK 938/17-infected bank vole and in four of ten Wistar rats via qPCR between 2 and 14 dpi. Interestingly, the infectious virus could be verified from those swab samples in only three individual bank voles. Between 5 and 7 dpi rather low titers of virus were excreted from bank voles (≥10^1.625^ TCID_50_ mL^−1^), while the highest virus titer was shed on day 9 post infection (10^2.6875^ TCID_50_ mL^−1^) (Figure 4).

Individual buccal swabs from bank voles inoculated with CPXV Ger2010MKY were qPCR-positive between 2 and 14 dpi. Four bank voles also shed infectious virus: low titers (≥10^1.625^ mL^−1^) were excreted between 7 and 12 dpi. The highest level of virus was excreted at day 12 post infection (10^2.75^ TCID_50_ mL^−1^) (Figure 4). Swabs of both bank voles that had to be euthanized due to >25% weight loss were tested positive for CPXV DNA (cycle quantification (Cq) values 29 and 31), but no virus could be isolated prior to euthanasia.

All swab samples taken from individual common voles tested negative for CPXV DNA regardless of the inoculated virus isolate.

#### 3.2.3. Viral Load in Organ Samples: CPXV Positive Turbinate Sample of a Bank Vole at 28 dpi

The distribution of OPV DNA in different organs sampled at 28 dpi was tested by qPCR. Organ samples from rodents inoculated with CPXV GerMygEK 938/17 revealed no viral DNA regardless of the animal species (bank vole, common vole, and Wistar rat). In contrast, after infection with CPXV Ger2010MKY euthanized bank voles (12 dpi) scored positive for infectious virus in turbinates (both 10^3.25^ TCID_50_ mL^−1^), and for viral DNA in the trachea (Cq values: 34 and 36), the skin (Cq values: 35 and 36), and the lung (Cq value: 32) of one bank vole. Organ samples from all other bank voles (28 dpi) were negative for OPV DNA except of one turbinate sample of a single animal. The CPXV titer of this sample reached 10^3.75^ TCID_50_ mL^−1^ (Table 2). 

#### 3.2.4. Serology: CPXV-Specific Seroconversion in Bank Voles with High Titers

Serum samples were obtained from every individual common vole and Wistar rat. Due to the sensitivity to anesthesia, we were able to achieve serum samples from eight of eleven individual bank voles (Table 3). In parallel, from all voles peritoneal lavage samples were collected.

Bank voles and Wistar rats inoculated with CPXV GerMygEK938/17 developed CPXV-specific antibodies with titers up to 1:640 (bank vole) or 1:1280 (Wistar rats), while no seroconversion was detectable in any of the inoculated common voles (Table 3). The sensitivity of the antibody detection in lavage samples was much lower—only in one of the bank vole samples antibodies were detected at a dilution of 1:20. 

Four bank voles infected with CPXV Ger2010MKY developed high CPXV-specific antibody titers up to 1:1280. From the remaining two bank voles, only peritoneal lavage was analyzed for antibodies and only one lavage sample reacted with an antibody titer of 1:20, while again no seroconversion was detectable in any of the inoculated common voles.

Overall, six of eight lavage samples from voles that showed seroconversion in the corresponding serum sample were reactive against CPXV with a low titer of 1:20 (Table 3). At the 1:40 dilution, only two CPXV-positive samples out of eight were observed (Table 3).

## 4. Discussion

Wild rodents, in particular voles (subfamily Arvicolinae), are presumed reservoir animals for CPXV maintenance, however, virus isolations from vole samples are extremely rare and most CPXV-isolates of today were generated from accidental host tissue samples. Human infections mostly happened because of the contact to diseased accidental hosts like cats or pet rats, while infections from a reservoir host to humans were not reported [38]. 

In 2015, the susceptibility of common voles for CPXV infection was demonstrated by experimental inoculations [27]. However, the role and importance of bank voles as reservoir hosts for CPXV is still a matter of debate. Our recent studies from 2017 have justified doubts on the susceptibility of bank voles as reservoirs for CPXV, because independent of the CPXV-isolate or infection route used, virus shedding was never detectable and only very few bank voles showed antibody titers above 1:80 [31]. However, a bank vole-derived isolate for experimental inoculation was not available at this time. 

In 2018, we successfully isolated the first bank vole-derived CPXV strain, GerMygEK 938/17 [32]. This new isolate provided the possibility to test the susceptibility of bank voles by experimental infection with a bank vole-derived strain. Nevertheless, growth kinetics of CPXV GerMygEK 938/17 on Vero76 cells in comparison to those of other CPXV strains like Brighton Red, RatPox09, or the very closely related CPXV strain Ger2010MKY allowed no proposition concerning the in vivo virulence. While all four tested CPXV strains showed a very similar outcome in the replication kinetics on Vero76 cells, the results of the animal experiments differed markedly [31]. Along with a previous study, we concluded that in vitro assays could not predict in vivo properties or virulence differences [39]. The same was observed in a recent study for the new CPXV strain GerMygEK 938/17 in comparison to Brighton Red and CPXV FM2292 on different cell lines (bank vole- and common vole-derived kidney cells and Vero76 cells) [32]. 

The here described animal experiments are to our knowledge the first comparing the infection of the most likely reservoir hosts, in particular bank vole and common vole with a bank vole-derived CPXV isolate and a very closely related CPXV strain originating from a cotton-top tamarin. As summarized in Table 4, bank voles inoculated with the bank vole-derived CPXV isolate GerMygEK 938/17 showed virus shedding from oropharyngeal secretions and a seroconversion rate of 100%. Interestingly, some bank voles inoculated with the most related strain CPXV Ger2010MKY even lost body weight of more than 25% and had to be euthanized.

Interestingly, not a single GerMygEK 938/17 inoculated bank vole showed any obvious clinical signs. However, animals from the wild are known to mask signs of disease as well as possible e.g., in the presence of human observers. This has to be taken into consideration for the evaluation of the absence of any clinical signs. It can be also speculated that sequence variation of the two related CPXV-like 3 strains in previously identified relevant genes like *vCCl*, *CrmB*, *CPXV0022*, and *CPXV194* are the reason of differences in virulence [32], and further studies will be necessary for clarification.

It needs to be highlighted that inoculated common voles exhibited neither virus shedding nor seroconversion. This is in clear contrast to the experimental infections of common voles with other CPXV strains, e.g., isolated from this vole species [27] (Table 4). The weight loss of the male animals might be explained due to their birth in the wild and the increased stress for these animals in captivity. The physical closeness to adult female voles without the possibility to mate has to be taken into consideration as well [40].

Wistar rats inoculated with the bank vole-derived isolate CPXV GerMygEK 938/17 exhibited no clinical signs, but all animals seroconverted. In former experiments, with the closely related cotton-top tamarin isolate CPXV Ger2010MKY, only two out of eight rats in the two different dose groups seroconverted [10]. In addition, the slight genetic differences might play a role.

The outcome of all these animal experiments further substantiates the recent hypothesis of reservoir species–specific phylogenetic lineages within the CPXV classification [31]. As depicted in Table 4, different CPXV strains display clearly different behavior in the different corresponding presumed reservoir animals and the Wistar rat animal model. While a common vole-derived CPXV isolate (CPXV-like 2 clade) leads to respiratory signs and prominent virus shedding in both the original reservoir animal and an animal model (Wistar rats) for an accidental host [27], the inoculated bank voles revealed no clinics, no virus shedding and a low titer seroconversion rate of 60% [31]. Vice versa, common voles inoculated with the bank vole-derived CPXV isolate (CPXV-like 3 clade) exhibited neither virus shedding nor seroconversion. Interestingly, in all inoculation experiments with different vole species and a series of different CPXV isolates, we could never observe any poxvirus lesions. This is only seen in accidental hosts and the rat model used here [3,4,5,6,7,8,9,10,11,29,30].

The basis of resistance or susceptibility of a mammal species for CPXV infection is determined by host characteristics [41,42] as well as viral factors [43]. An example is the outcome of ECTV infection in laboratory mice: resistance and susceptibility are controlled by genetic factors of the host and are associated with multiple immune response mechanisms [44]. However even C57/BL6 mice, resistant to infection, respond serologically to ECTV inoculation [45]. 

To further support the hypothesis of a reservoir host specificity or preference of diverse CPXV clades, we will need more animal experiments with CPXV-like 3 isolates both in bank voles and in common voles. Furthermore, more detailed studies reflecting the genetic differences between CPXV-like 2 clade and CPXV-like 3 clade and the immune response (innate and adaptive) of the different hosts versus the different CPXV. 

An additional conclusion from both the phylogenetic data and the in vivo characterization is that bank voles possibly also mediated the emergence of the CPXV Ger2010MKY isolate in the cotton-top tamarin holding in Thuringia, Germany, 2010 [10], although the low number of trapped rodents a few months later tested negative for CPXV. 

In addition, Wistar rats as the accidental host model are susceptible for an infection with clear clinical signs including respiratory signs and virus shedding when inoculated with viruses of the common vole-related CPXV-like 2 clade, but are much more resistant to the bank vole-related isolates of CPXV-like 3 clade or ECTV [46]. This is also matching the observation that CPXV strains from the bank-vole related clade were only in very few cases isolated from accidental hosts [10]. 

A possible explanation for the differences between both CPXV clades could be the fact that the gene *CPXV0030* is missing in the genome of CPXV-like 3 isolates. We were recently able to demonstrate the impact of gene *CPXV0030* on the virulence in Wistar rats [47]. The gene encodes the CPXV 7-transmembrane G protein-coupled receptor-like protein (g7tGP) [48]. Further experiments with knock-out/knock-in mutants are conceivable just as infection experiments with isolates of CPXV-like 1 lineage, where the *g7tGP* gene is also missing. 

In summary, the experimental inoculation of bank voles with isolates of CPXV-like 3 clade provided very important pieces of the puzzle about competent reservoir species of CPXV in central Europe. In addition to the sequence data-based polyphyletic character of the “species” CPXV [49], the “clades” of CPXV might also rely on different reservoir host preferences and adaptations.

## Figures and Tables

**Table 1:** Design of the animal experiments.

Cowpox Virus (CPXV)			Animals		Experiment Design				
Phylogenetic Lineage	Strain	Origin	Species	Number of Animals per Group	Application Route	Dose of Inoculum/ Animal	Duration of the Experiment	Nasal/buccal Swabs	Study
CPXV-like 3	GerMygEK 938/17	Bank vole	Bank vole	5*	oronasal	10^5.5^ TCID_50_	28 dpi	Every 2^nd^ day	This study
			Common vole	6	oronasal	10^5.5^ TCID_50_	28 dpi	Every 2^nd^ day	This study
			Wistar rat	10	oronasal	10^5.5^ TCID_50_	28 dpi	Every 2^nd^ day	This study
CPXV-like 3	Ger2010MKY	Cotton-top tamarin	Bank vole	6	oronasal	10^5.5^ TCID_50_	28 dpi	Every 2^nd^ day	This study
			Common vole	6	oronasal	10^5.5^ TCID_50_	28 dpi	Every 2^nd^ day	This study
			Wistar rat	4	oronasal	10^4^/10^6^ TCID_50_	28 dpi	Every 2^nd^ day	[10]

dpi, days post inoculation; TCID_50_, Tissue Culture Infectious Dose 50 %. *One additional animal died unrelated during anesthesia.

**Table 2:** Viral DNA detection and virus titers in different organs.

CPXV Strain	Species	Dissection	Tissue						
				Turbinate	Trachea	Lung	Liver	Spleen	Skin
**GerMygEK 938/17**	Bank vole	28 dpi		0/5*	0/5	0/5	0/5	0/5	0/5
	Common vole	28 dpi		0/6	0/6	0/6	0/6	0/6	0/6
	Wistar rat	28 dpi		0/10	0/10	0/10	0/10	0/10	0/10
**Ger2010MKY**	Bank vole	12 dpi^#^		2/2	2/2	1/2	0/2	0/2	2/2
			Cq value (TCID_50_ mL^−1^)	24.8 (10^3.25^) 26.3 (10^3.25^)	34.2 36.1	32.2			35.1 36
		28 dpi		1/4	0/4	0/4	0/4	0/4	0/4
			Cq value (TCID_50_ mL^−1^)	22.9 (10^3.75^)					
	Common vole	28 dpi		0/6	0/6	0/6	0/6	0/6	0/6

Cowpox virus, CPXV; dpi, days post inoculation; TCID_50_, Tissue Culture Infectious Dose 50 %. * Number of animals tested viral genome positive per total tested animals. ^#^ Two animals were euthanized at 12 dpi because of weight loss of more than 25%.

**Table 3:** Seroconversion of CPXV-inoculated rodents at 28 days post inoculation.

CPXV Strain	Species	Number of Animals per Group	Number of Serum/Lavage Samples	Dilution							
				1:20	1:40	1:80	1:160	1:320	1:640	1:1280	1:2560
				seroreactive	seropositive						
GerMygEK 938/17	Bank vole	5^*^	Serum 4 Lavage 5	4/4 1/5^#^	4/4 0/5^#^	4/4	4/4	3/4	2/4	0/4	0/4
	Common vole	6	Serum 6 Lavage 6	0/6 0/6	0/6 0/6						
	Wistar rat	10	Serum 10	10/10	10/10	10/10	10/10	10/10	10/10	6/10	0/10
Ger2010MKY	Bank vole	6	Serum 4 Lavage 6	4/4 5/6^##^	4/4 2/6^##^	4/4	4/4	4/4	2/4	1/4	0/4
	Common vole	6	Serum 6 Lavage 6	0/60/6	0/6 0/6						

Cowpox virus, CPXV; *One additional animal died unrelated during anesthesia. # Peritoneal lavage of all animals was tested. Only one bank vole sample, already positive in the serum, tested positive. ## Peritoneal lavage of all animals was tested. Lavage of both bank voles without parallel serum sample reacted with a titer of 1:20.

**Table 4:** Comparison of different CPXV Strains and Reservoirs.

Cowpox Virus (CPXV)			Animals Inoculated		Results of Animal Experiments					Study
	Phylogenetic Lineage	Strain / Origin	Species	Dose of Inoculum/ Animal	Clinical Signs	Virus Shedding	Viral DNA in Organs (28 dpi)	Seroconversion	Mortality	
**Common vole reservoir**	CPXV-like 2	FM2292 / Common vole	Common vole	10^4^ TCID_50_	none	2/3	none	66%	0%	[27]
				10^6^ TCID_50_	respiratory	3/3	1/3 (nasal septum)	100%	33%	[27]
			Bank vole	10^5^ TCID_50_	none	none	none	60%	0%	[31]
			Wistar rat	10^4^ TCID_50_	Respiratory Pox-like lesions	4/4	none	100%	0%	[27]
				10^6^ TCID_50_	Respiratory Pox-like lesions	3/3	none	100%	0%	[27]
**Bank vole reservoir**	CPXV-like 3	GerMygEK 938/17 / Bank vole	Bank vole	10^5.5^ TCID_50_	none	5/5	none	100%	0%	This study
			Common vole	10^5.5^ TCID_50_	none	none	none	0%	0%	This study
			Wistar rat	10^5.5^ TCID_50_	none	none	none	100%	0%	This study
		Ger2010MKY / Cotton-top tamarin	Bank vole	10^5.5^ TCID_50_	Weight loss up to 25%	6/6	1/6 (nasal septum)	83%	33%	This study
			Common vole	10^5.5^ TCID_50_	none	none	none	0%	0%	This study
			Wistar rat	10^4^ TCID_50_	none	none	none	25%	0%	[10]
				10^6^ TCID_50_	none	none	none	25%	0%	[10]

dpi, days post inoculation; TCID_50_, Tissue Culture Infectious Dose 50.
